# Metabolomic Analyses Reveal That IAA from *Serratia marcescens* Lkbn100 Promotes Plant Defense during Infection of *Fusarium graminearum* in Sorghum

**DOI:** 10.3390/plants13162184

**Published:** 2024-08-07

**Authors:** Jichen Yan, Nawei Qi, Jing Xu, Lan Hu, Yu Jiang, Yuanjun Bai

**Affiliations:** 1 Institute of Plant Protection, Liaoning Academy of Agricultural Sciences, Shenyang 110161, China; yjc891013@163.com (J.Y.); mljasmine2004@163.com (J.X.); laner_lnnky@163.com (L.H.); 2 College of Life Sciences, Shenyang Normal University, Shenyang 110034, China; nwqi199208@163.com; 3 Institute of Rice, Liaoning Academy of Agricultural Sciences, Shenyang 110161, China

**Keywords:** sorghum root rot, *Fusarium graminearum*, biocontrol bacteria, metabolomics analysis, indole-3-acetic acid (IAA), induced resistance

## Abstract

Global sorghum production has been significantly reduced due to the occurrence of sorghum root rot caused by the fungus *Fusarium graminearum*. The utilization of biocontrol microorganisms has emerged as an effective strategy. However, the underlying mechanisms remain unclear. Therefore, the aim of this study was to investigate the effectiveness of biocontrol bacteria in inducing sorghum resistance against sorghum root rot and explore the potential induced resistance mechanisms through metabolomics analysis. The results revealed that the biocontrol bacteria Lnkb100, identified as *Serratia marcescens* (GenBank: PP152264), significantly enhanced the resistance of sorghum against sorghum root rot and promoted its growth, leading to increased seed weight. Targeted metabolomics analysis demonstrated that the highest concentration of the hormone IAA (indole-3-acetic acid) was detected in the metabolites of Lnkb100. Treatment with IAA enhanced the activity of disease-related enzymes such as SOD, CAT, POD and PPO in sorghum, thereby improving its resistance against sorghum root rot. Further untargeted metabolomic analysis revealed that IAA treatment resulted in higher concentrations of metabolites involved in the resistance against *F. graminearum*, such as geniposidic acid, 5-L-Glutamyl-taurine, formononetin 7-O-glucoside-6″-O-malonate, as well as higher concentrations of the defense-related molecules volicitin and JA. Additionally, “secondary bile acid biosynthesis” and “glycerophospholipid metabolism” pathways were found to play significant roles in the defense response of sorghum against fungal infection. These findings provide a reliable theoretical basis for utilizing biocontrol microorganisms to control sorghum root rot.

## 1. Introduction

Sorghum, as one of the world’s top five cereals, plays a crucial role in global food and feed security China [1]. Sorghum has a long and widespread history of cultivation, serving as a vital crop for food and feed, as well as a raw material for brewing and vinegar production [2]. However, sorghum growth is often hindered by various biotic and abiotic stresses, among which sorghum root rot caused by *Fusarium graminearum* is a significant biotic factor. Traditional chemical control methods have raised concerns due to their negative environmental impacts, highlighting the need for alternative biocontrol agents. Plant growth-promoting rhizobacteria (PGPR), known for their ability to enhance plant growth and produce natural compounds that protect against diseases, have emerged as promising biocontrol agents [3]. *Lysobacter enzymogenes* inhibits *Fusarium* growth by producing antibiotics, while lactobacillus acidophilic, *lactobacillus plantarum*, *lactobacillus fermented*, *lactobacillus cheeseweed* and *Lactobacillus brevis*, found in honey, suppress Fumonisin-producing *Fusarium* growth. Additionally, isolates of *Streptomyces* and *Trichoderma* have shown effective control against *Fusarium* [4,5]. Bioagents derived from Aspergillus, fluorescent Pseudomonas, and Bacillus have demonstrated fruitful results in preventing pineapple top rot caused by *Fusarium* [6].

Plant hormones are small signaling molecules that regulate plant development and adaptive responses to various environmental stresses. Indole-3-acetic acid (IAA) is widely recognized as a pivotal plant growth regulator, with its signaling pathways being involved in plant disease resistance [7]. More than 80% of rhizobacteria harbor the ability to produce and secrete plant-like growth hormones, including nitrogen-fixing spirillum, nitrogen-fixing bacteria, *Escherichia coli*, *Pseudomonas aeruginosa* or *Staphylococcus* [8]. PGPR-synthesized auxins are known to promote plant growth directly or indirectly by releasing tryptophan and auxin-like compounds into the rhizosphere. For instance, auxins support the formation of lateral and adventitious roots, enhancing mineral absorption and exudate production, thereby fostering bacterial proliferation [9]. Additionally, PGPR-derived auxins exhibit the ability to mitigate detrimental effects from various environmental stresses such as drought, salinity, or soil pollution [10,11,12,13]. Notably, exogenous auxin treatments have demonstrated positive effects in inducing disease resistance in plants [14,15].

Despite extensive research into the role of auxins in plant diseases and defense responses, the mechanism underlying exogenous auxin-induced sorghum diseases and resistance against *Fusarium* infection remains unclear. Accordingly, this study aims to investigate the metabolomic intervention of sorghum-induced resistance to disease and *Fusarium* infection using IAA produced by biocontrol bacteria. Additionally, by shedding light on the potential mechanisms of defense responses induced by biocontrol bacteria, this research focuses on novel theoretical approaches and practical advancements in the safe and efficient management of sorghum diseases.

## 2. Results

### 2.1. Identification of Serratia marcescens Isolate LnKb100

**Figure 1:**
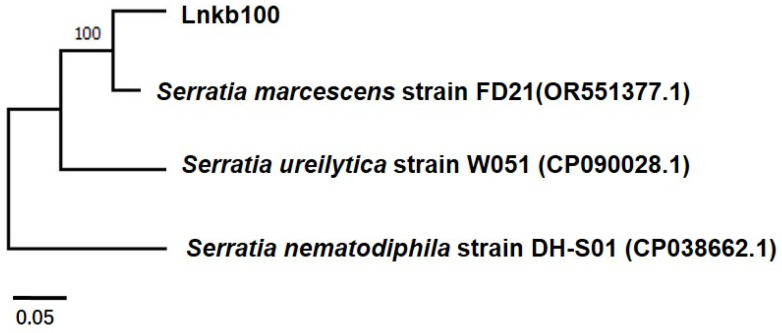
Phylogenetic tree of biocontrol bacteria phylogenetic tree, based on the DNA sequences of the 16S rRNA gene of selected strains encoding the biocontrol bacteria. The bootstrap values (%) displayed on the branches were calculated from 1000 replicates.

The results of the greenhouse pot experiments revealed significant differences between sorghum treated with Lnkb100 and the untreated control. Additionally, treated sorghum exhibited significant differences in plant height, root length, stem weight, and fresh root weight compared to the control group (Table 1). The field trials further supported these findings, demonstrating that sorghum roots treated with Lnkb100 had significantly reduced disease severity compared to the controls. In 2022 and 2023, substantial field control efficacy was observed at the trial fields of the Liaoning Academy of Agricultural Sciences (41.811797 N, 123.549373 E) and the Crop Research Institute of Liaoning (41.265037 N, 123.146486 E), with protection rates of 68 and 54.84% in 2022, and 53.33 and 57.14% in 2023, respectively (Table 2). Moreover, the hundred-grain weight of sorghum treated with Lnkb100 was significantly greater than that of the control group, resulting in notable increases in production in the following years. Specifically, production enhancements of 9.01% and 16.8% were recorded in 2022, and 12.5% and 14.46% in 2023, for the respective test fields (Table 3). To gain further insights into the potential biocontrol mechanisms of Lnkb100, the bacterium was identified through analysis of the 16S rRNA gene. The results were Lnkb100 *Serratia marcescens* (GenBank: PP152264) (Figure 1).

### 2.2. Impact of IAA-Rich Metabolites from Biocontrol Bacterium Lnkb100 on Plant Health and Disease Management

**Figure 2:**
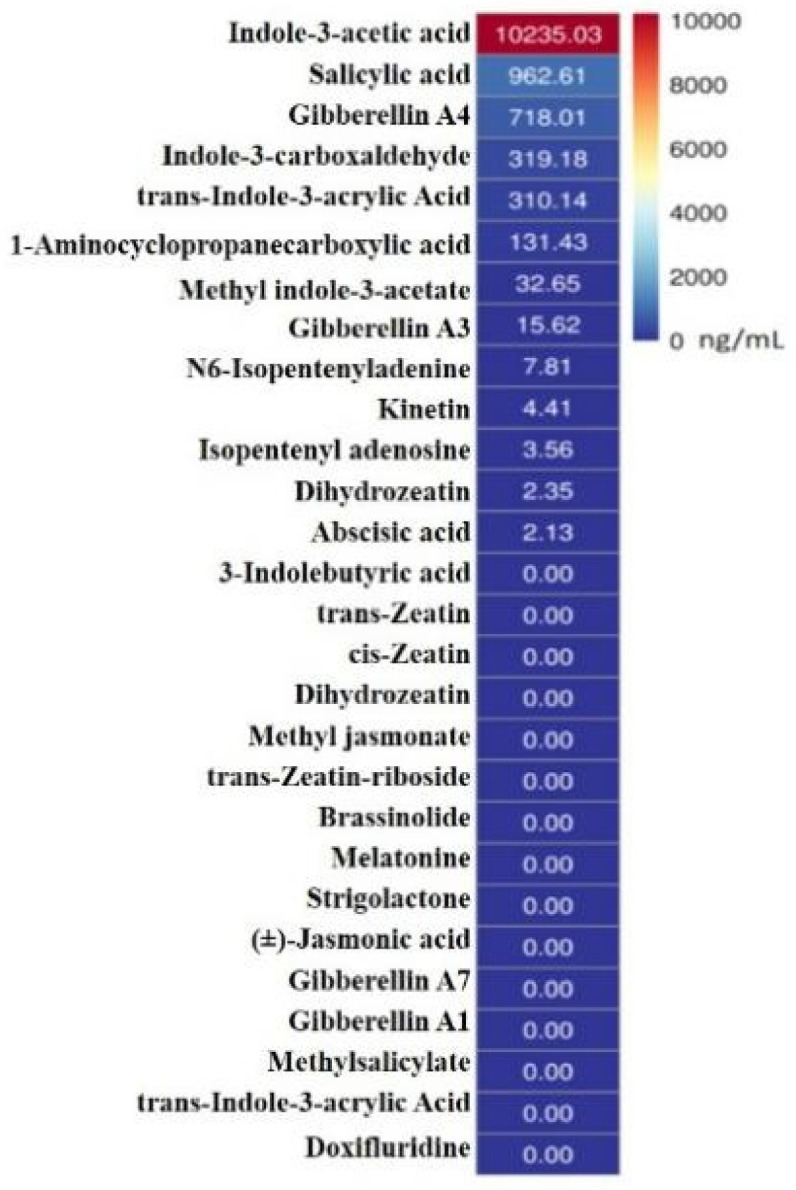
Types and content of hormones in the metabolites of the biocontrol bacterium Lnkb100.

**Figure 3:**
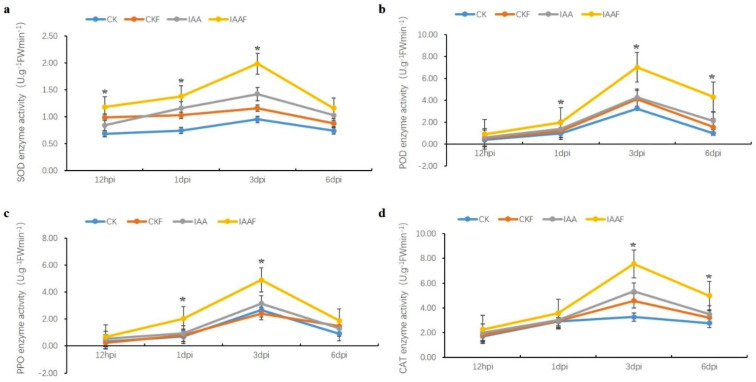
Enzyme-modulating effects of IAA in the metabolites of biocontrol bacteria. Note: CK: blank control; IAA: IAA treatment only; CKF: *Fusarium* inoculation only; IAAF: IAA treatment followed by *F. graminearum*. inoculation; Asterisks indicate significant differences (* *p* < 0.05). Figures (**a**–**d**) respectively illustrate the SOD, POD, PPO, and CAT-modulating effects of IAA on the metabolites of biocontrol bacteria.

To determine the composition and levels of hormones present in the metabolites of Serratia marcescens Lnkb100, this study employed the UHPLC-MRM-MS/MS platform to target the detection of hormonal components within Lnkb100 metabolites. The analysis revealed a complex profile of plant hormones, with a predominance of auxins, gibberellins, cytokinins, and their derivatives. Specifically, indole-3-acetic acid (IAA) emerged as the most abundant hormone, present at a concentration of 10,235.03 ng/mL, indicating its pivotal role in the metabolic profile of Lnkb100. Other notable auxins included salicylic acid and indole-3-carboxaldehyde, detected at concentrations of 962.61 ng/mL and 319.18 ng/mL, respectively. Among the gibberellins, gibberellin A4 was identified at a concentration of 718.01 ng/mL, while gibberellin A3 was present at a significantly lower concentration of 15.62 ng/mL. The cytokinins, represented by trans-zeatin and its derivatives, were found to be virtually absent, with concentrations near the detection limit (0 ng/mL). Additionally, abscisic acid was detected at a concentration of 2.13 ng/mL, and jasmonic acid was not detected in the metabolite profile. This comprehensive analysis underscores the rich hormonal composition of Lnkb100, with IAA being the dominant component, which may have significant implications for its biocontrol properties (Figure 2). The application of IAA from Lnkb100 metabolites to sorghum seeds not only promoted plant growth but also suppressed *Fusarium* root rot (Table 4). The impact of different concentrations of Indole-3-acetic acid (IAA) on sorghum was evaluated, and significant variations were observed, especially at concentrations between 1 and 0.1 μg/mL. At the 0.1 μg/mL concentration, the pot experiment demonstrated an 80.00% disease prevention rate, which was markedly higher than that of the control group. Sorghum treated with 0.1 μg/mL IAA exhibited significant increases in plant height, root length, stem weight and fresh root weight compared to the controls. These results suggest that the application of exogenous IAA at an appropriate concentration (0.1 μg/mL) enhances resistance to *Fusarium* root rot in sorghum and promotes growth. Furthermore, this study discovered that 0.1 μg/mL IAA could regulate the activity of disease resistance-related enzymes in sorghum (Figure 3). Compared to other treatments, the application of IAA to sorghum significantly enhanced the activities of superoxide dismutase (SOD), catalase (CAT), polyphenol oxidase (PPO), and peroxidase (POD). The peak enzyme activity occurred on the third day post-infection (3 dpi), indicating that the optimal timing for treatment (3 dpi) and the IAA concentration (0.1 μg/mL) were selected for further studies.

### 2.3. Analysis of the Metabolomic Characteristics of All Samples

**Figure 4:**
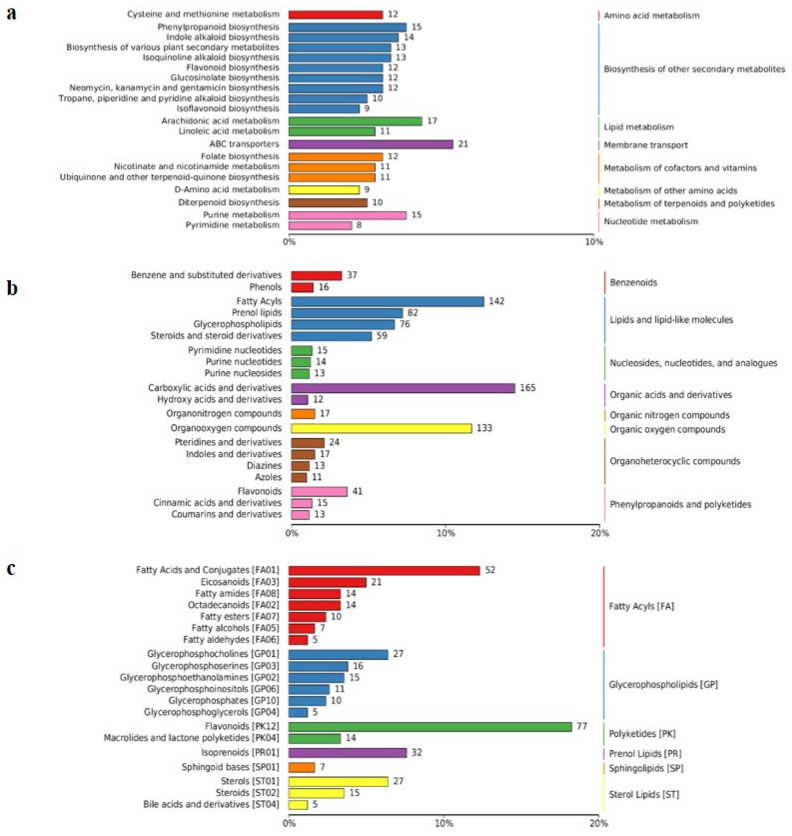
Enrichment heatmaps of metabolomic analysis. (**a**) KEGG Database Classification Summary. (**b**) HMDB Database Classification Summary. (**c**) LIPID MAPS Database Classification Summary. Entries within the same box represent hierarchical classification information, corresponding to the super class and class information in the respective databases. The length of the bars represents the number of metabolites annotated in each classification.

**Figure 5:**
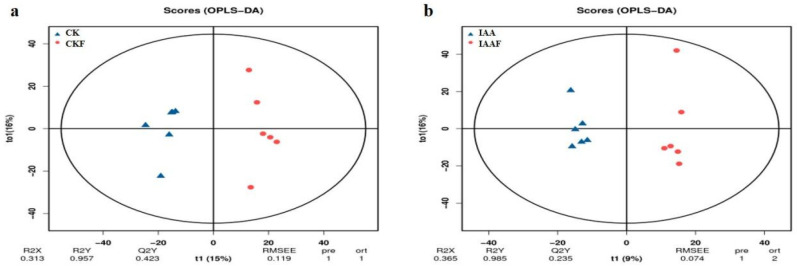
OPLS-DA model analysis was performed using the most diverse sample. (**a**). OPLS-DA in CK vs. CKF group. (**b**). OPLS-DA in IAA vs. IAAF group. Note: R2X: Model’s explanatory power for input matrix X; R2Y: Model’s explanatory power for output matrix Y; Q2Y: Model’s predictive ability, closer to 1 indicates stability and reliability; Model Effectiveness: Q2Y > 0.5 indicates an effective model; Model Excellence: Q2Y > 0.9 indicates an excellent model, and a more stable and reliable model, meaning that this model can be used to screen differential metabolites.

**Figure 6:**
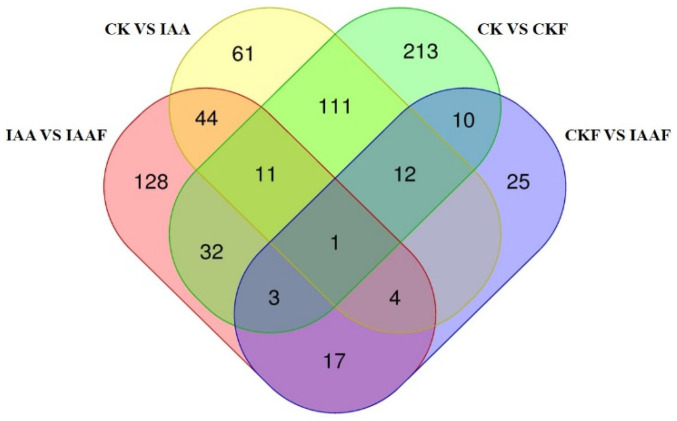
Venn Diagram of differential metabolites between different treatment groups. Each circle in the diagram represents a comparison group, and the numbers in the overlapping regions represent the number of shared differential metabolites between the comparison groups. The numbers outside the overlapping regions represent the number of unique differential metabolites for each comparison group.

In order to understand the metabolic characteristics of the samples, various databases were used in this study to classify and enrich the metabolomic profiles of 24 samples. Using the Kyoto Encyclopedia of Genes and Genomes (KEGG) database (https://www.genome.jp/kegg/pathway.html, accessed on 1 January 2023) classification and enrichment analysis were performed and the top 20 pathways were enriched, including “Biosynthesis of other sensory system”, ”Metabolism of cofactors and vitamins”, ”Lipid metabolism”, “Nucleotide metabolism”, ”Membrane transport”, ”Amino acid metabolism”, and “Metabolism of terpenoids and polyketides”, etc. In addition, ”Membrane transport” in “ABC transporters”, “Arachidonic acid metabolism” in “Lipid metabolism” and “Purine metabolism” in “Biosynthesis of other sensory system” were the most significantly enriched pathways (Figure 4a). Using the HMDB database (https://hmdb.ca/metabolites, accessed on 1 January 2023), classification and summation were performed. The top 20 pathways were enriched, including “Lipids and lipid-like molecules”, ”Organic acids and derivatives”, ”Organic oxygen compounds”, ”Phenylpropanoids and polyketides”, ”Nucleotides, nucleotides, and analogues”, ”Benzenoids”, ”Organoheterocyclic compounds”, and “Organic nitrogen compounds”, etc. In addition, ”Carboxylic acids and derivatives” in “Organic acids and derivatives”, “Fatty acyls” in “Lipids and lipid-like molecules”, ”Prenol lipids” in “Prenol lipids”, and ”Flavonoids” in “Phenylpropanoids and polyketides” were the most significantly enriched pathways (Figure 4b). Using the LIPID Maps database (http://www.lipidmaps.org/), classification and summation were performed. The top 20 pathways were enriched, including “Fatty acyls [FA]”, ”Glycerolipids [GL]”, “Glycerophospholipids [GP]”, ”Polyketides [PK]”, ”Prenol Lipids [PR]”, ”Sphingolipids [SP]” and “Sterol Lipids [ST]”. In addition, ”Flavonoids [PK12]” in “Polyketides [PK]”, ”Fatty Acids and Conjugates [FA01]”, ”Fatty Acids and Conjugates [FA01]”, ”Eicosanoids [FA03]” in “Fatty acyls [FA]”, ”Isoprenoids [PR01]” in “Prenol Lipids [PR]”, ”Glycerophosphocholines [GP01]” in “Glycerolipids [GL]” and ”Sterols [ST01]” in “Sterol Lipids [ST]” were the most significantly enriched pathways (Figure 4c). An OPLS-DA model was used with the variable importance in the projection (VIP) statistics of the first principal component (threshold > 1) and the *p* value of the Student’s *t*-test (threshold < 0.05) to select important variables responsible for the separation of groups. The metabolic profiles of 24 samples were analyzed and significant differences in the metabolic composition were found between CK and CKF and between IAA and IAAF (Figure 5). In addition, the differentially expressed metabolites between different treatments showed significant differences (Figure 6). After being separately treated with disease (CKF) or IAA, more differentially expressed substances or up-regulated substances were expressed in the sorghum treated with disease alone than in the sorghum treated with IAA alone, and the most differentially expressed substances were in the sorghum treated with disease alone. Interestingly, there were significantly more differentially expressed substances in IAA and IAAF than in CKF and IAA, and there were the least differentially expressed substances in CKF and IAAF, with only 42 up-regulated substances. These substances are the focus of the next analysis (Table 5).

### 2.4. Metabolites with Significant Differential Expression in Metabolomic Analysis

**Figure 7:**
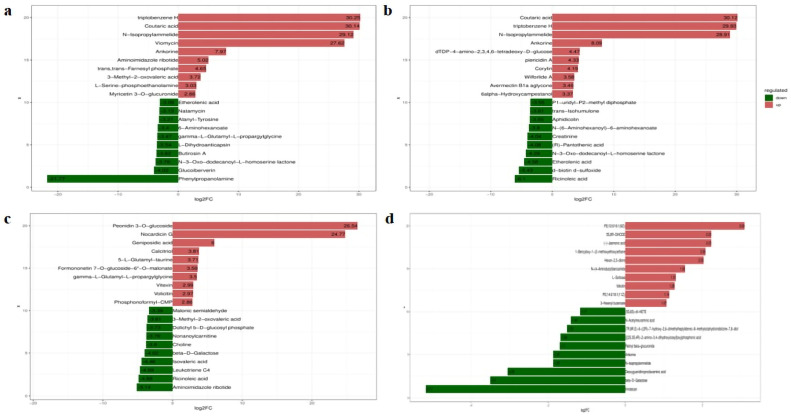
Bar graph of differential metabolite fold change. Figure (**a**) represents the comparison between CK and IAA treatments. Figure (**b**) represents the comparison between CK and CKF treatments. Figure (**c**) represents the comparison between IAA and IAAF treatments. Figure (**d**) represents the comparison between CKF and IAAF treatments. The labels on each bar indicate the names of the metabolites, with up-regulated metabolites shown in red and down-regulated metabolites shown in green. The length of each bar represents the log fold change (log FC). Only the top 10 up-regulated and down-regulated differential metabolites with the largest fold change are displayed.

To identify the specific differentially expressed metabolites between different treatments, a bar graph displaying the top and bottom 10 differentially expressed metabolites with the largest differences multiples was utilized. After inoculation with *F. graminearum* alone (CK vs. CKF), the up-regulated differentially expressed metabolites in the sorghum metabolism pathways included Coutaric acid, triphenylethane H,N-Isopropylammelide, Ankorine, dTDP-4-amino-2,3,4,6-tetradeoxy-D-glucose, piericidin A, Corylin, Wilforlide A, Avermectin B1a aglycone, and 6-alpha-hydroxycampestanol. The down-regulated differentially expressed metabolites in the metabolic pathway included ricinoleic acid, d-biotin d-sulfoxide, etherolenic acid, N-3-oxo-dodecanoyl-L-homoserine lactone, (R)-pantothenic acid, creatinine, N-(6-aminohexanoyl)-6-aminohexanoate, aphidicolin, trans-isohumulone, and P1-uridyl-P2-methyl diphosphate (Figure 7b). After treatment with IAA alone (CK vs. IAA), the up-regulated differentially expressed metabolites in the sorghum metabolism pathways included triphenylethane H, Coutaric acid, N-Isopropylammelide, Viomycin, Ankorine, Aminoimidazole ribotide, trans-farnesyl phosphate,3-methyl-2-oxovaleric acid, L-Serine-phosphoethanolamine and Myricetin 3-O-glucuronide. The down-regulated differentially expressed metabolites in the metabolic pathway included phenylpropanolamine, glucoiberverin, N-3-oxo-dodecanoyl-L-homoserine lactone, butirosin A, L-Dihydroanticapsin, gamma-L-glutamyl-L-propargylglycine, 6-aminohexanoate, alanyl-tyrosine, natamycin, and etherolenic acid (Figure 7a). When comparing the treatment of *Fusarium* after IAA treatment and no *Fusarium* treatment (IAA vs. IAAF), the up-regulated differentially expressed metabolites included Peonidin 3-O-glucoside, Nocardicin G, Geniposidic acid, Calcitriol, 5-L-glutamyl-taurine, Formononetin 7-O-glucoside-6″-O-malonate, Gamma-L-glutamyl-L-propargylglycine, Vitexin, Volicitin and Phosphonoformyl-CMP. The down-regulated differentially expressed metabolites included Aminoimidazole ribotide, Ricinoleic acid, Leukotriene C4, Isovaleric acid, beta-D-Galactose, Choline, Nonanoylcarnitine, Dolichyl b-D-glucosyl phosphate, 3-methyl-2-oxovaleric acid and Malonic semialdehyde (Figure 7c). When comparing the treatment of IAA before *Fusarium* treatment and no IAA treatment (CKF vs. IAAF), the up-regulated differentially expressed metabolites included PE(12:0/16:1(9Z), 5S,8R-Dihydrodeoxyadenosine, (-)-Jasmonic acid, 1-Benzyloxy-1-(2-methoxyethoxy)ethane, Hexan-2,3-dione, N-(4-Aminobutyl)benzamide, L-Sorbose and Volicitin. The down-regulated differentially expressed metabolites included Irinotecan, beta-D-Galactose, Deoxyguanidinoproclavaminic acid, N-Isopropylammelide, Ankorine, Retinyl beta-glucuronide, [(2S,3S,4R)-2-amino-3,4-dihydroxyicosyl]oxy]phosphonic acid, (7R,8R, E)-6-((2R)-7-hydroxy-2,6-dimethylheptylidene)-8-methyloctahydroindolizine-7,8-diol, and N-Acetylneuraminic acid and (5S,6S)-di-HETE (Figure 7d). These differentially expressed metabolites provide valuable insights into the metabolic changes induced by different treatments and further highlight the specific metabolic pathways involved in each treatment.

### 2.5. Metabolic Pathway Enrichment Analysis

**Figure 8:**
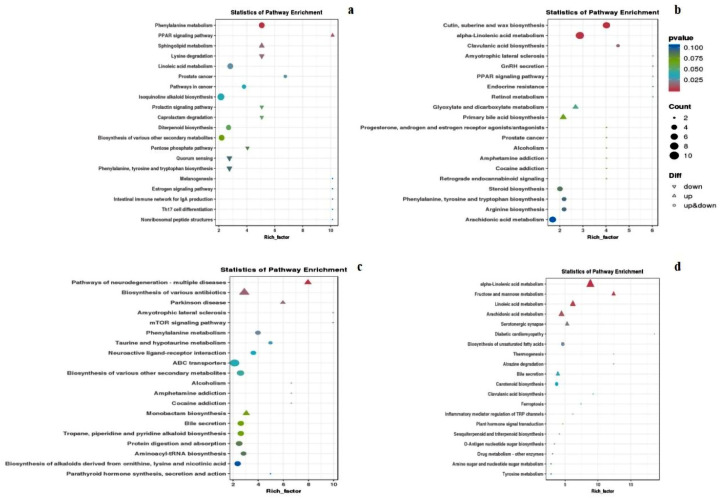
Dot plot of differential metabolite KEGG pathway enrichment, comparing different treatments. (**a**) The comparison between CK and IAA treatments. (**b**) The comparison between CK and CKF treatments. (**c**) The comparison between IAA and IAAF treatments. (**d**) The comparison between CKF and IAAF treatments. Each dot represents a KEGG pathway, with the x-axis indicating the enrichment factor. The y-axis represents pathway names. Dot color represents the *p* value, and the circle size indicates the number of enriched differential metabolites. The dot shape indicates up-regulated or down-regulated status. Pathways closer to the upper right corner have a higher reference value.

To explore the metabolic pathways associated with the differentially expressed metabolites between different treatments, KEGG_pathway_enrich dotplot was utilized to visualize the 20 most significantly enriched metabolic pathways. After *Fusarium* treatment alone (CK vs. CKF), the differentially expressed metabolites in the sorghum metabolic pathways, such as Glyoxylate and dicarboxylate metabolism and Primary bile acid biosynthesis, were up-regulated. Conversely, the metabolites in cutin, suberine and wax biosynthesis, alpha-linolenic acid metabolism, clavulanic acid biosynthesis, steroid biosynthesis, phenylalanine, tyrosine and tryptophan biosynthesis, arginine biosynthesis and the arachidonic acid metabolism pathways were both up-regulated and down-regulated (Figure 8b). After IAA treatment alone (CK vs. IAA), the differentially expressed metabolites in the sorghum metabolic pathways, such as the PPAR signaling pathway and Sphingolipid metabolism, were up-regulated. In contrast, the metabolites in lysine degradation, the prolactin signaling pathway, caprolactam degradation, the pentose phosphate pathway, quorum sensing, and phenylalanine, tyrosine and tryptophan biosynthesis were down-regulated. The differentially expressed metabolites in phenylalanine metabolism, linoleic acid metabolism, prostate cancer, pathways in cancer, isoquinoline alkaloid biosynthesis, diterpenoid biosynthesis, and the biosynthesis of various other secondary metabolites pathways were both up-regulated and down-regulated (Figure 8a). In the comparison of IAA treatment followed by *Fusarium* treatment and no IAA treatment (IAA vs. IAAF), the differentially expressed metabolites in the pathways of neurodegeneration, multiple diseases, the biosynthesis of various antibiotics, Parkinson’s disease, and monobactam biosynthesis were up-regulated. Conversely, the metabolites in phenylalanine metabolism, taurine and hypotaurine metabolism, neuroactive ligand–receptor interaction, ABC transporters, bile secretion, tropane, piperidine and pyridine alkaloid biosynthesis, protein digestion and absorption, aminoacyl-tRNA biosynthesis and the biosynthesis of alkaloids derived from ornithine, lysine and nicotinic acid were both up-regulated and down-regulated (Figure 8c). In the comparison of *Fusarium* treatment before IAA treatment and no IAA treatment (CKF vs. IAAF), the differentially expressed metabolites in alpha-linolenic acid metabolism, arachidonic acid metabolism, linoleic acid metabolism, fructose and mannose metabolism, serotonergic synapse, and bile secretion, plant hormone signal transduction pathways were up-regulated. On the other hand, the metabolites in the biosynthesis of unsaturated fatty acids were both up-regulated and down-regulated (Figure 8d). These metabolic pathway enrichment analyses shed light on the potential metabolic alterations and pathways involved in the different treatments.

## 3. Discussion

*Fusarium* root rot of sorghum is caused by various pathogenic fungi, which can spread to healthy seedlings through spores or mycelia [16]. Biological control has been proposed as a sustainable and environmentally friendly strategy to mitigate plant diseases and establish sustainable agricultural systems. The main objective of biological control is to utilize antagonistic organisms to suppress the incidence of plant pathogen disease. In our study, we aimed to investigate the efficacy of Lnkb100, identified as *Serratia marcescens* by the molecular analysis of 16SrRNA, for its biological control against *Fusarium* root rot in sorghum. Greenhouse and field experiments were conducted to evaluate the impact of Lnkb100 on *F. graminearum* growth inhibition, as well as the promotion of sorghum growth and yield. *S. marcescens* has been found to possess various beneficial functions, including phosphate solubilization, indole-3-acetic acid production, siderophore synthesis, and plant remediation [17,18,19,20,21]. Moreover, *S. marcescens* has been reported to have direct or indirect inhibitory effects against different *Fusatrium* species [22,23,24,25].

Metabolomics techniques provide valuable insights into the complex regulatory networks involved in host resistance and the modulation of plant metabolic pathways in response to plant diseases, making them highly applicable in sustainable agriculture. To explore the defense mechanisms of biocontrol bacteria against *Fusarium*, we employed targeted metabolomics analysis based on LC-MS to examine the types and contents of hormones in the metabolome of the biocontrol bacteria *Serratia marcescens* Lnkb100. Our analysis identified indole-3-acetic acid (IAA) as the most abundant hormone in the metabolic profile of Lnkb100. Treatment with IAA not only enhanced sorghum resistance to *Fusarium* but also promoted sorghum growth. Similarly, the exogenous application of auxin has been shown to play a positive role in plant disease control, as plants treated with exogenous auxin exhibit enhanced resistance to diseases [26,27]. Additionally, we conducted a non-targeted metabolomics analysis based on LC-MS to investigate the differential metabolites and metabolic pathways involved in the IAA treatment of sorghum in response to *Fusarium* infection. Using the KEGG, HMDB and LIPID MAPS databases, we observed the significant enrichment of “lipid metabolism” and “phenylpropanoid and polyketide compounds” in the sorghum metabolome.

It is worth noting that the number of differentially expressed metabolites in the IAA treatment group was lower compared to the control group, indicating the significant impact of IAA induction or *Fusarium* infection on the up-regulation of specific metabolites. These metabolites included lipids and lipid-like molecules such as 5S, 8R-DiHODE; (-)-Jasmonic acid, PE(14:0/18:1(11Z)), PE(12:0/16:1(9Z)), Geniposidic acid, Calcitriol, 6alpha-Hydroxycampestanol, Wilforlide A and Volicitin. Flavonoids such as Corylin, Formononetin 7-O-glucoside-6″-O-malonate, Peonidin 3-O-glucoside and Vitexin were also observed. Furthermore, organic acids and derivatives (e.g., 3-Methyl-2-oxovaleric acid, 5-L-Glutamyl-taurine, L-Serine-phosphoethanolamine), organic oxygen compounds (e.g., Aminoimidazole ribotide, L-Sorbose, 1-Benzyloxy-1-(2-methoxyethoxy)ethane), terpenoids (e.g., trans, trans-Farnesyl phosphate), macrolides (e.g., Avermectin A2a aglycone), peptides (e.g., Viomycin) and monobactams (e.g., Nocardicin G) were identified as significantly up-regulated metabolites. Previous studies have indicated the involvement of these metabolites in plant metabolism and their response to various stresses, including biological and non-biological stresses. Specific compounds such as 6alpha-Hydroxycampestanol, Coutaric acid and Corylin have demonstrated inhibitory effects on *Fusarium* growth, providing protection against *Fusarium* diseases [24,25,28,29,30,31,32,33]. Furthermore, natural compounds produced by biocontrol bacteria, such as Avermectin A2a aglycone, 3-Methyl-2-oxovaleric acid and Nocardicin G, have been identified as potential agents for seed treatment to control *Fusarium* and promote plant growth and yield [34,35,36,37,38]. Geniposidic acid and 5-L-Glutamyl-taurine, which are significantly up-regulated only in response to *Fusarium* infection in sorghum, exhibit antioxidant, antibacterial and antifungal activities, particularly against *Fusarium* and other pathogenic fungi [39,40,41]. During the interaction between plants and *Fusarium*, plant antioxidants are biosynthesized in pathways involved in the production of antifungal toxins [42,43]. Volicitin (N-((17-hydroxylinolenoyl)-L-glutamine)) induces corn resistance and acts as a lure in the presence of *Fusarium* [44]. The application of this chemical to tobacco induces jasmonic acid (JA) and JA-isoleucine (JA-ILE) coupling [45], thereby activating defense genes in insects through the production of fatty acid–amino acid conjugates (FACs) mediated by JA [46,47]. In our study, we observed the significant enrichment of the “Linoleic acid metabolism” and “alpha-linolenic acid metabolism” pathways in the roots of IAA-treated sorghum, as well as in the roots of IAA-treated sorghum responding to *Fusarium*. Additionally, the “Bile secretion” and “Plant hormone signal transduction” pathways were significantly enriched only in the roots of IAA-treated sorghum responding to *Fusarium*. These findings indicate the important roles played by the “Linoleic acid metabolism”, “alpha-linolenic acid metabolism” “Bile secretion” and “Plant hormone signal transduction” pathways in the IAA-induced resistance of sorghum to *Fusarium*. Our observations support existing evidence and suggest that these bioactive organic compounds can be employed to enhance the resistance of major crops to economically significant pathogens in the future.

## 4. Materials and Methods

### 4.1. Molecular Identification of Biocontrol Bacteria

The selected strain Lnkb100 was identified by analyzing its 16S rRNA gene sequence. DNA Extraction: Bacterial DNA was extracted from 1.5 mL of fermentation broth using the Fast TIANamp Bacteria DNA Kit, as per the manufacturer’s protocol provided by TIANGEN (Beijing, China). 16S rDNA Amplification: The complete 16S rDNA was amplified via PCR with the universal bacterial primers 27F (5′-AGA GTT TGA TCC TGG CTC AG-3′) and 1492R (5′-ACG GCT ACC TTG TTA CGA CTT-3′). PCR Mixture: The reaction mixture comprised 1 μL of template DNA (50 ng/μL), 1 μL each of forward and reverse primers (10 μM), 10 μL of 2× Taq PCR master mix from TIANGEN (Beijing, China), and 7 μL of double-distilled water. PCR Protocol: The PCR program followed the methodology reported by Kumar et al. (2015) [42] and was executed on a Bio-Rad PCR thermal cycler located in Hercules, California, USA. PCR Product Analysis: The resulting PCR products were analyzed by electrophoresis on 1.2% (*w*/*v*) agarose gels stained with Goldview (Real-Times Biotechnology, Beijing, China). The gels were then sent to Dingguo Gene Co., Ltd., Beijing, China, for sequencing. Sequence Analysis: The high-quality sequences obtained were analyzed using BLASTn (NCBI; http://blast.ncbi.nlm.nih.gov, accessed on 1 January 2023) to confirm the identity of each bacterium.

### 4.2. Microorganisms and Plant Samples

Highly pathogenic *Fusarium graminearum* isolates were obtained as single-spore pure cultures from 274 sorghum grain samples collected from 13 provinces and 17 cities in China. The isolates were identified and confirmed as *F. graminearum*, the causal agent of sorghum root rot disease. The strains were stored at the Plant Protection Research Institute, Liaoning Academy of Agricultural Sciences, China.

The biocontrol bacterium Lnkb100, which promotes the strong induction of resistance against *Fusarium graminearum* root rot in sorghum, was selected from a large number of soil bacteria strains (Supplementary Figure S1). The strain is stored at the General Microbiological Center of the China General Microbiological Culture Collection Center (CGMCC) under the registration number CGMCC No. 27118.

Sorghum seeds BT × 623 provided by the Sorghum Research Institute, Liaoning Academy of Agricultural Sciences, China, were used in this study.

### 4.3. Field and Laboratory Experiments

*Serratia marcescens* isolate Lnkb100 was inoculated on nutrient agar (NA) medium in test tubes, followed by incubation in nutrient broth medium. The fermentation was carried out at 25–28 °C with shaking at 150 rpm for 2–3 days. After the fermentation, a cell suspension at a concentration of 1.0 × 10^9^ CFU mL^−1^ was used for the sorghum seed treatment in the laboratory and field experiments.

#### 4.3.1. Laboratory Experiment

The cell suspension of *S. marcescens* isolate Lnkb100 was fermented at a concentration of 1.0 × 10^9^ CFU mL^−1^. Subsequently, 1 mL of this cell suspension was employed to coat 30 g of seeds. The coated sorghum seeds were air-dried and sown in 12 × 12 cm seedling pots. Each pot was inoculated with 10 mL of *Fusarium graminearum* macroconidia spore suspension (10 × 10^8^ CFU mL^−1^). Uncoated sorghum seeds (CK) and sorghum seeds coated with NA liquid medium (NA) were used as control treatments. After 45 days of growth, the disease severity was assessed for each treatment, and the plant height (cm), root length (cm), fresh root weight (g) and stem weight (g) were also measured.

#### 4.3.2. Field Experiment

Field efficacy experiments were conducted in the experimental fields of the Liaoning Academy of Agricultural Sciences (41.811797 N,123.549373 E) and Liaoning Crops Institute (41.265037 N, 123.146486 E) in 2022 and 2023, respectively. Both fields were severely affected by sorghum root rot, with sorghum and maize as the main crops. The cell suspension was used for the seed coating section of the laboratory experiment. Uncoated sorghum seeds (CK) and sorghum seeds coated with NA liquid medium (NA) were used as control treatments. After 45 days of growth, the severity of the sorghum root rot was assessed by uprooting the sorghum plants while keeping the roots intact. The disease severity was evaluated, and the disease control efficacy (%) was calculated. After 180 days of growth, the hundred-grain weight (g) was measured by harvesting and drying the sorghum panicles, and the yield increase effect (%) was calculated.

### 4.4. Hormone Detection in Biocontrol Bacteria

To detect the content of 28 hormones in the biocontrol bacteria, ultra-high-performance liquid chromatography–triple quadrupole mass spectrometry (UHPLC-MRM-MS/MS) analysis was conducted in this study. Specifically, a Waters ACQUITY I-Class UHPLC system equipped with an ACQUITY UPLC HSS T3 (100 × 2.1 mm, 1.8 μm, Waters, Shanghai, China) column was used for the chromatographic separation of the target compounds. The mobile phase consisted of 0.1% formic acid in water (phase A) and 0.1% formic acid in acetonitrile (phase B). The column temperature was set at 40 °C, and the sample tray temperature was set at 10 °C. The injection volume was 5 μL. The analysis was performed using a SCIEX QTRAP 6500+ triple quadrupole mass spectrometer (Sciex, Framingham, MA, USA) equipped with an IonDrive Turbo V ESI ion source (Sciex, Framingham, MA, USA) in multiple reaction monitoring (MRM) mode. The ion source parameters were set as follows: Curtain Gas = 35 psi, IonSpray Voltage = +5500 V/−4500 V, Temperature = 550 °C, Ion Source Gas 1 = 50 psi, Ion Source Gas 2 = 55 psi. Prior to UHPLC-MS/MS analysis, standard solutions of the target compounds were introduced into the mass spectrometer. For each target compound, several precursor-to-product ion pairs (transitions) with the highest signal intensity were selected, and the MRM parameters were optimized. The most responsive ion pairs were used for quantitative analysis, while the other ion pairs were used for qualitative analysis of the target compounds.

### 4.5. IAA Regulated Sorghum Resistant Fusarium

#### 4.5.1. Promotion of Growth and Disease Resistance by IAA in Laboratory Experiment

Different concentrations of solutions were prepared for the seed treatment. Different concentrations of indole 3-acetic acid solutions (IAA, Sigma Aldrich, St. Louis, MO, USA), namely 1000, 100, 10, 1 and 0.1 mL/g, were used to coat the seeds at 1 mL for 30 g of seeds. The coated sorghum seeds were air-dried and sown in 12 × 12 cm seedling pots. Each pot was inoculated with 10 mL of *F. graminearum* macroconidia spore suspension (10 × 10^8^ CFU mL^−1^).

Uncoated sorghum seeds were used as the control (CK). After 45 days of growth, the disease severity was assessed for four treatments: blank control (CK), IAA treatment only (IAA), *Fusarium* inoculation only (CKF) and IAA treatment followed by *Fusarium* inoculation (IAAF). The plant height (cm), root length (cm), fresh root weight (g) and stem weight (g) were measured. The most effective concentration of IAA was selected for the assay of disease-related enzyme activity.

#### 4.5.2. Regulation of Enzymes Activity by IAA in Sorgum Artifically Inoculated with *Fusarium graminearum*

The sorghum seeds were coated with the concentration of IAA solution at a ratio of 1:30 (mL:g). The coated seeds were air-dried and sown in 12 × 12 cm seedling pots. Each pot was inoculated with *Fusarium graminearum* spore suspension. Uncoated sorghum seeds (CK) were used as the control. At 12 h post-inoculation (12 hpi), 1 day post-inoculation (1 dpi), 3 days post-inoculation (3 dpi), and 6 days post-inoculation (6 dpi), the sorghum roots were collected for enzyme activity detection using colorimetric methods and specific assay kits (all kits were provided by Sangon Biotech Co., Ltd., Shanghai, China):

The Superoxide Dismutase (SOD) activity was measured at 560 nm using the SOD Activity Assay Kit (product code D799593-0050).

The Peroxidase (POD) activity at 470 nm was measured using the POD Activity Assay Kit (product code D799591-0050).

The Polyphenol Oxidase (PPO) activity at 410 nm was measured using the PPO Activity Assay Kit (product code D799595-0050).

The Cinnamyl Alcohol Dehydrogenase (CAD) activity at 340 nm was measured using the CAD Activity Assay Kit (product code D799095-0050).

For detailed protocols, please refer to the following links:

SOD Activity Assay Kit:

[Product Detail] (https://store.sangon.com/productDetail?productInfo.code=D799593, accessed on 1 January 2024).

POD Activity Assay Kit:

[Product Detail] (https://store.sangon.com/productDetail?productInfo.code=D799591, accessed on 1 January 2024).

PPO Activity Assay Kit:

[Product Detail] (https://store.sangon.com/productDetail?productInfo.code=D799595, accessed on 1 January 2024).

CAD Activity Assay Kit:

[Product Detail] (https://store.sangon.com/productDetail?productInfo.code=D799095, accessed on 1 January 2024).

### 4.6. Metabolomic Analysis

IAA (0.1 mg/L) was used for seed treatment. The sorghum seeds were coated with the above-mentioned IAA concentrations at a ratio of 1:30 (ml:g). The coated seeds were air-dried and sown in 12 × 12 cm seedling pots. Each pot was inoculated with 10 mL of *Fusarium graminearum* spore suspension (10 × 10^8^ CFU mL^−1^). Uncoated sorghum seeds were used as the control (CK). At 3 dpi, the sorghum roots were collected and stored in liquid nitrogen. These samples were then transported to Biomarker Technologies and subjected to metabolomic analysis using the BMKCloud platform (www.biocloud.net).

Sample preparation: The freeze-dried roots were crushed using a mixer mill and mixed with a methanol–water solution for overnight incubation to extract metabolites. Centrifugation and filtration: The extracts were centrifuged, adsorbed on carbon-GCB SPE columns, and filtered through SCAA-104 filters. Liquid chromatography–mass spectrometry (LC-MS) analysis (reference): The effluent was connected to an ESI-triple quadrupole-linear ion trap (QTRAP)-MS for LIT and triple quadrupole (QQQ) scans (reference). Instrument tuning and mass calibration: Instrument tuning and mass calibration were performed using a polyethylene glycol solution. MRM experiments and data processing: Multiple reaction monitoring (MRM) experiments were conducted in both the QQQ and LIT modes to monitor specific MRM channels. The mass spectrometry data were processed using analysis software. Identification and quantification of metabolites: Mass spectrometry was used for the qualitative and quantitative analysis of the metabolites in the samples, with reference to the Kyoto Encyclopedia of Genes and Genomes (KEGG) database (https://www.genome.jp/kegg/pathway.html, accessed on 1 January 2024), the Human Metabolome Database (HMDB) (https://hmdb.ca/metabolites, accessed on 1 January 2024), and the LIPID Maps database (http://www.lipidmaps.org/) for local metabolite measurement databases.

### 4.7. Statistical Analysis

The data in this study were obtained from at least three independent determinations and presented as the mean ± standard deviation (SD). One-way analysis of variance (ANOVA) was used for the comparison of the datasets, followed by Tukey’s test for statistical analysis. A *p*-value less than 0.05 was considered statistically significant.

Principal component analysis (PCA) and orthogonal projections to latent structures–discriminant analysis (OPLS-DA) were used for data analysis, model construction, and model validation. Differential metabolites were screened based on the criteria of VIP > 1 and *p*-value < 0.05 for the differences in products between the control group and the experimental group. KEGG enrichment analysis was performed to analyze the significantly different metabolomics products by searching the KEGG database.

## 5. Conclusions

In conclusion, this study provides valuable insights into the molecular mechanisms underlying the biocontrol activity of *Serratia marcescens* Lnkb100 against *Fusarium* root rot in sorghum. Through targeted metabolomics analyses, we identified IAA as a key hormone produced by Lnkb100, which enhances resistance and promotes the growth of sorghum. Additionally, through non-targeted metabolomics analyses, we discovered specific metabolites that are differentially produced in response to IAA induction and *Fusarium* infection, including lipids, flavonoids, organic acids, terpenoids, macrolides, peptides and monobactams. The “Linoleic acid metabolism”, ”alpha-linolenic acid metabolism”, “Bile secretion” and “Plant hormone signal transduction” pathways were found to be related in the IAA-induced resistance of sorghum to *Fusarium*. Our findings contribute to a better understanding of the potential application of *Serratia marcescens* Lnkb100 as a biological control agent for *Fusarium* root rot and pave the way for further research in this field.

## Figures and Tables

**Table 1:** Potting efficacy of Lnkb100 fermented product coating treatment on sorghum seeds.

	Plant Heigh (cm)	Root Length (cm)	Stem Weight (g)	Root Weight (g)	Disease Severity	Potting Effectiveness %
CK	35.79 ± 0.56 a	5.84 ± 0.59 a	8.75 ± 0.24 a	1.78 ± 0.16 a	5.8 ± 0.43 a	--
NA	34.46 ± 0.46 b	5.42 ± 0.63 b	6.60 ± 0.28 b	1.41 ± 0.26 b	4.6 ± 0.38 b	20.69
Lnkb100	32.05 ± 0.37 b	5.77 ± 0.68 b	6.82 ± 0.16 b	1.95 ± 0.42 c	2.4 ± 0.25 c	58.62

Note: mean ± standard deviation. Different letters following the data in the same column indicate significant differences at the level of *p* < 0.05 (Tukey’s test).

**Table 2:** Field efficacy of Lnkb100-coated treatment on sorghum seeds.

Treatment	2022				2023			
	Site 1		Site 2		Site 1		Site 2	
	Disease Severity	Efficacy (%)	Disease Severity	Efficacy (%)	Disease Severity	Efficacy (%)	Disease Severity	Efficacy (%)
CK	5.00 ± 0.23 a	--	6.20 ± 0.21 a	--	6.00 ± 0.34 a	--	4.20 ± 0.18 a	--
NA	4.00 ± 0.16 b	20.00	5.40 ± 0.39 b	12.90	5.00 ± 0.47 b	16.67	3.80 ± 0.24 b	9.52
Lnkb100	1.60 ± 0.27 c	68.00	2.80 ± 0.18 c	54.84	2.80 ± 0.43 c	53.33	1.80 ± 0.38 c	57.14

Note: data mean ± standard deviation. Different letters following the data in the same column indicate significant differences at the level of *p* < 0.05 (Tukey’s test).

**Table 3:** Field yield of sorghum seeds treated with Lnkb100 fermentation product coating.

Treatment	2022				2023			
	Site 1		Site 2		Site 1		Site 2	
	Hundred-Grain Weight (g)	Yield Increase (%)	Hundred-Grain Weight (g)	Yield Increase (%)	Hundred-Grain Weight (g)	Yield Increase (%)	Hundred-Grain Weight (g)	Yield Increase (%)
CK	2.33 ± 0.21 a	0.00	2.38 ± 0.25 a	0.00	2.24 ± 0.13 a	0.00	2.42 ± 0.24 a	0.00
NA	2.34 ± 0.32 a	0.43	2.37 ± 0.36 a	−0.42	2.28 ± 0.12 a	1.79	2.43 ± 0.23 a	0.41
Lnkb100	2.54 ± 0.12 b	9.01	2.78 ± 0.21 b	16.81	2.52 ± 0.15 b	12.50	2.77 ± 0.12 b	14.46

Site 1: Liaoning Academy of Agricultural Sciences. Site 2: Liaoning Institute of Economic Crops. Different letters following the data in the same column indicate significant differences at the level of *p* < 0.05 (Tukey’s test).

**Table 4:** Pot experiment of maize seeds treated with Indole-3-Acetic Acid (IAA).

	Plant Heigh (cm)	Root Length (cm)	Stem Weight (g)	Root Weight (g)	Disease Severity	Potting Effectiveness %
CK	34.57 ± 0.69 a	6.14 ± 0.62 a	8.67 ± 0.21 a	1.20 ± 0.16 a	5.00 ± 0.33 c	--
IAA1000	40.53 ± 0.75 b	7.51 ± 0.27 c	9.22 ± 0.28 b	1.68 ± 0.24 c	2.33 ± 0.33 b	45.34
IAA100	46.97 ± 0.82 c	8.60 ± 0.81 d	8.79 ± 0.24 b	1.71 ± 0.21 c	2.33 ± 0.13 b	45.34
IAA10	46.10 ± 0.51 c	6.40 ± 0.13 b	8..77 ± 0.42 a	1.58 ± 0.37 b	2.33 ± 0.13 b	45.34
IAA1	40.00 ± 0.67 b	7.97 ± 0.29 c	9.62 ± 0.25 c	1.57 ± 0.12 b	1.00 ± 0.00 a	80.00
IAA0.1	41.83 ± 0.59 c	8.93 ± 0.37 d	9.83 ± 0.38 c	1.45 ± 0.23 b	1.00 ± 0.00 a	80.00

Note: The results were obtained after a 45-day investigation. The data in the table represent the mean ± standard error/deviation. Different letters following the data in the same column indicate significant differences at the level of *p* < 0.05 (Duncan’s new multiple range test).

**Table 5:** Number of differential metabolites in each group.

Group	Total	Up	Down
CK vs. IAA	2346	90	154
CK vs. CKF	2346	221	172
IAA vs. IAAF	2346	135	105
CKF vs. IAAF	2346	42	30

## Data Availability

Data are contained within the article.

## Supplementary Materials

The following supporting information can be downloaded at: https://www.mdpi.com/article/10.3390/plants13162184/s1, Figure S1: Efficacy of Lnkb100 in Controlling Fusarium in Pot; Table S1: Fusarium Separation Ratio in Sorghum Seeds from Different Regions.
