# Supplementary material for: Metabolomic Analyses Reveal That IAA from Serratia marcescens Lkbn100 Promotes Plant Defense during Infection of Fusarium graminearum in Sorghum
Source: Plants (Basel). 2024 Aug 7;13(16):2184. doi: 10.3390/plants13162184 (PMC11360247; doi:10.3390/plants13162184)
Supplement: Supplementary file 1 [file plants-13-02184-s001.zip › plants-2957958-supplementary.pdf]

plants

**Supplementary Table S1.** Fusarium Separation Ratio in Sorghum Seeds from Different Regions.

| Region | Fusarium Separation Ratio (%) |
|---|---|
| Shanxi Province | 1.26 |
| Inner Mongolia Autonomous Region | 1.33 |
| Hebei Province | 2.00 |
| Heilongjiang Province | 2.76 |
| Jilin Province | 3.71 |
| Guizhou Province | 4.22 |
| Hunan Province | 9.63 |
| Hubei Province | 10.81 |
| Liaoning Province | 29.74 |
| Gansu Province | 30.03 |
| Sichuan Province | 40.44 |
| Shandong Province | 43.73 |
| Henan Province | 43.87 |

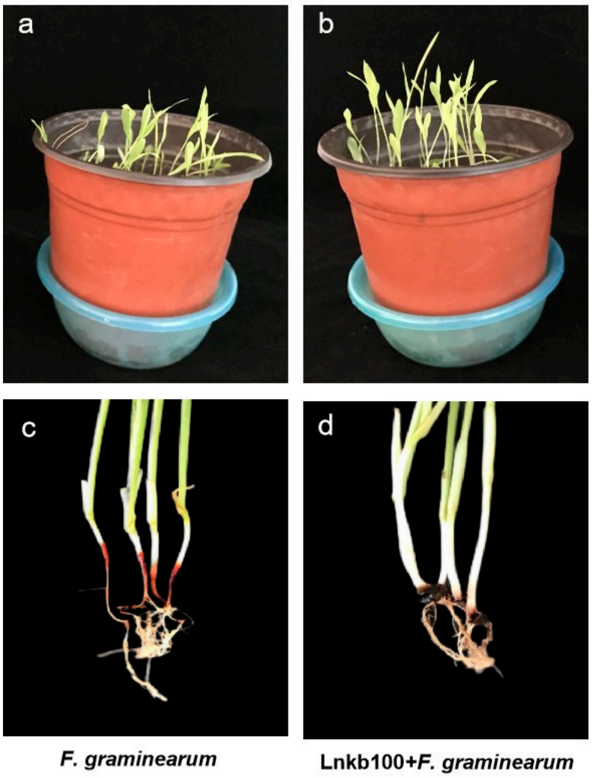


**Supplementary Figure S1.** Efficacy of Lnkb100 in Controlling Fusarium in Pot.
